# Effectiveness of inspector mechanism for the emergency infection prevention and control in the SARS-CoV-2 epidemic period: a self-control real-word study

**DOI:** 10.1186/s12879-023-08682-2

**Published:** 2023-12-06

**Authors:** Yu Lv, Qian Xiang, Xiaoyan Jiang, Bo Zhang, Jiayu Wu, Hongrong Cao

**Affiliations:** 1 Healthcare-Associated Infection Control Center, Sichuan Provincial People’s Hospital, school of medicine, University of Electronic Science and Technology of China, Chengdu, 610072 Sichuan P. R. China; 2 Development Department, Chengdu Yiou Technology Co. LTD, Chengdu, 610000 Sichuan P. R. China

**Keywords:** COVID-19 pandemic, SARS, Infection prevention and control, Inspector mechanism

## Abstract

**Background:**

To ensure emergency infection prevention and control (IPC) can be fully supervised and monitored in coronavirus disease (COVID-19) epidemic period, a three-level inspector mechanism called "Internal self-check, Departmental cross-check, and Verification of outstanding key and difficult issues" was established in southwest China. The present study aimed to explore the effectiveness of inspector mechanism for the emergency IPC.

**Methods:**

A self-control real-world study was conducted during COVID-19 epidemic period from 2020 to 2022. An innovative designed mobile phone application was used to realize paperless information transmission and data management. Data were compared between inspection levels using SPSS 19.0 software.

**Results:**

A total of 2,800,132 supervision records were collected, including 149,137 comprehensive epidemic IPC projects, 1,410,093 personal protective equipment (PPE) use, 1,223,595 wearing and removing process of PPE and 17,307 ultraviolet light-detectable fluorescent (UV/F) surface marker. During the study period, the inspectors and subjects explored many optimized IPC measures. The compliance rate of check items has exceeded 98%, and internal self-check has a statistically significant higher rate than departmental cross-check (99.95% versus 98.74%, *χ*^2^ = 26111.479, *P* < 0.001). Compare with the failure rate in internal self check, the failure rate of PPE usage and wearing/removing process was statistically higher in departmental cross-check (*χ*^2^ = 1957.987, *P* < 0.001, *χ*^2^ = 465.610, *P* < 0.001, respectively). The overall clearance rate of UV/F surface markers is 87.88%, but there is no statistically significant difference over the three years of the present study (*F* = 2.902, *P* = 0.071).

**Conclusions:**

Inspector mechanism for the emergency IPC completed an incredible inspection workload and offered creative assistance to combat the COVID-19 outbreak. These methods and accumulated experiences should be helpful for us to strengthen IPC for future epidemic.

**Supplementary Information:**

The online version contains supplementary material available at 10.1186/s12879-023-08682-2.

## Background

The global pandemic of coronavirus disease (COVID-19) created a public health crisis in many countries which was caused by the novel severe acute respiratory syndrome coronavirus 2 (SARS-CoV-2) [1, 2]. From 2020 to 2022, the COVID-19 epidemic has changed the world dramatically due to multiple epidemic peaks of mutant variants such as Alpha, Beta, Gamma, Delta and Omicron [3]. At the time of this writing, there have been 767,750,853 confirmed cases of COVID-19, including 6,941,095 deaths, reported to World Health Organization (WHO) [4]. In the wake of so much loss, there is an opportunity to maintain gains made during the COVID-19 epidemic and strengthen the infection prevention and control (IPC) for future epidemic and pandemic response efforts [5, 6]. However, with COVID-19 over as a global health emergency declared by WHO on May 5, 2023, many tested excellent IPC strategies may face the risk of being unconsciously abandoned or forgotten, such as inspector mechanism [7].

The inspector mechanism, as a reasonable supervision and management strategy, has demonstrated various forms and connotations around the world during the COVID-19 crisis [8]. Personal protective equipment (PPE) related inspector mechanism was most commonly structured to monitor or optimize PPE utilization, such as “PPE Spotters” in Chicago and “PPE police” in Kuwait [9, 10]. In some special scenarios, the inspector mechanism has been extensively used in various aspects related to IPC. A learning-practice-examination-supervision model practiced in Shanghai's Fangcang shelter hospitals has shown an excellent protective effect of infection control for third-party personnel [8]. In negative pressure isolation wards of Guangdong Second Provincial General Hospital, an observing system was established to maintain the normal operation of wards, supervise the implementation of disinfection and ensure a PPE sufficient supply [11]. Of note, the inspector mechanism became a nationally recommended strategy after the observing system in Guangdong Province was recognized by the Health and Family Planning Commission of China (FPCC). We performed a three-level inspector mechanism in such occasion to supervise and inspect the strengthened measures on the basis of daily IPC, so as to avoid healthcare-associated infections (HAIs) caused by COVID-19 [12, 13]. As the shortage of full-time staff in HAI management is common in China's tertiary hospitals [14], such as Sichuan Provincial People's Hospital which has 4,130 beds, 5,480 in-service medical workers and more than 1,300 intern students, but only 12 full-time HAI management staff, we attempted to recruit more part-time staff to establish an enforcement strategy through the three-level inspector mechanism to make strong supervision and monitoring for the emergency IPC of COVID-19. In the present study, we performed a self-control real-word study to explore the effectiveness of inspector mechanism for the emergency IPC in COVID-19 epidemic period from 2020 to 2022.

## Material and methods

### Study design

A real-world study was conducted in Sichuan Academy of Medical Sciences, Sichuan People's Hospital, School of Medicine, University of Electronic Science and Technology of China, a tertiary care teaching hospital in Chengdu in the region of Sichuan (Western China). The informed consent was waived by the local ethics committee as it was an observational study.

All 116 departments and units of the hospital were included in this intervention study, including 9 intensive care units (ICUs), 74 general clinical departments, 17 outpatient and emergency units, 9 medical technology departments and 7 auxiliary departments. SM Table S1 in the Supplement presents the details of intervention units.

### Study protocol

**Fig. 1:**
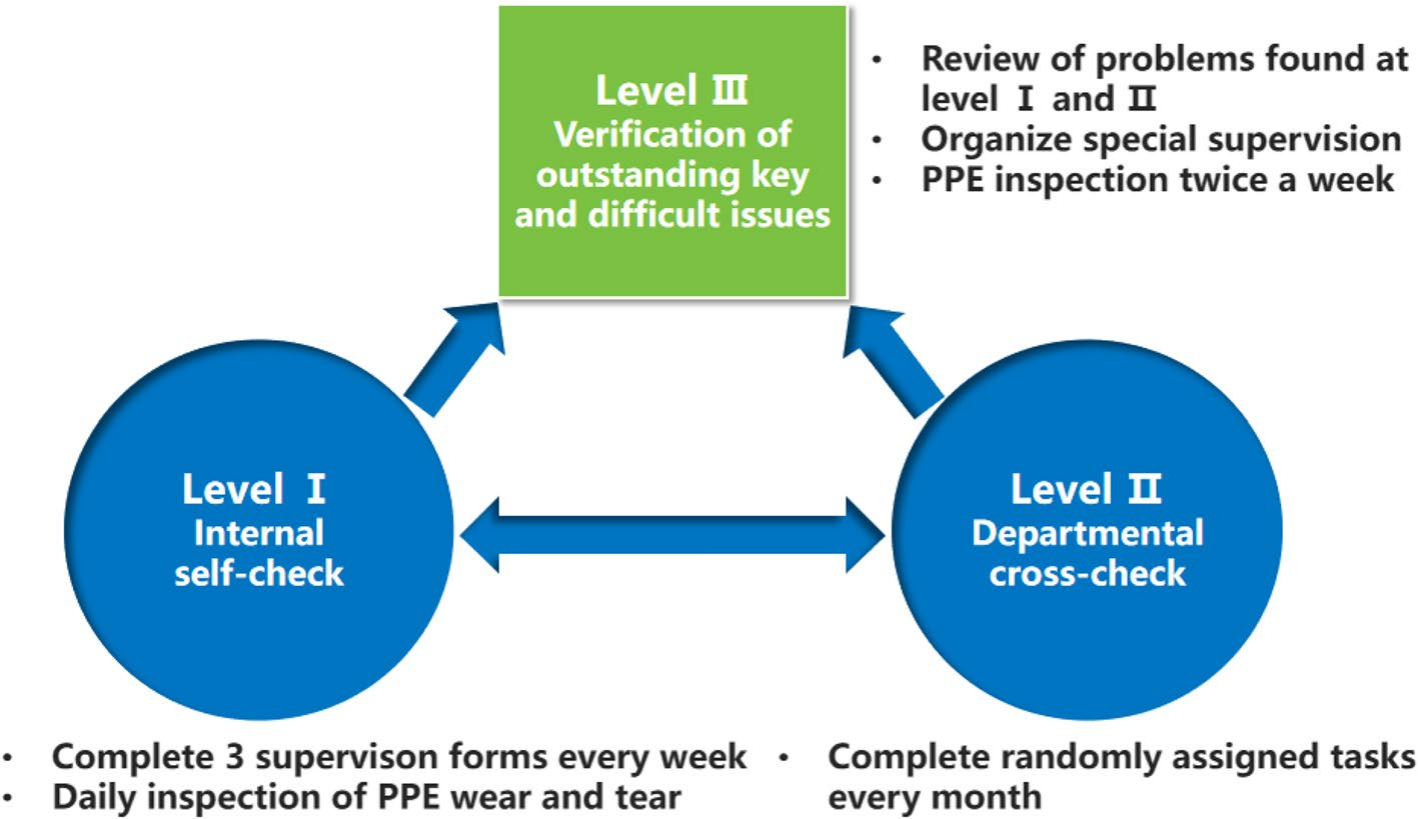
The part-time inspector system for COVID-19 prevention and control. This system includes Internal self-examination, Departmental cross-examination, and Verification of outstanding key and difficult issues

A three-level inspector mechanism, called "Internal self-check, Departmental cross-check, and Verification of outstanding key and difficult issues" (Fig. 1), was established in March 2020, with the vice-president of the hospital as the team leader, to make up for the lack of full-time HAI management staff, and to ensure each emergency IPC measures can be fully supervised and monitored. The emergency IPC, matched with the COVID-19 emergency response, is based on the Guidelines for the COVID-19 IPC in medical institutions issued by the National Health Commission, including the emergency plan and workflow, training, emergency isolation ward, cleaning and disinfection, nucleic acid sampling, medical staff protection, pre-inspection triage, strict hand hygiene, medical waste disposal, medical fabric disposal, strict management of accompanying and visiting, boiling water room management and self-symptoms and nucleic acid monitoring [15–17].

The regulations and implementation rules of inspector mechanism, signed by the hospital legal representative, documents the system's organizational framework, responsibilities, guarantee, implementation, coordination and operational details, and places special emphasis on the personal interests of inspectors, including ① the heads of each department should provide inspectors with half a day off routine work per week to complete the inspection tasks full-time; ② allowances are supplemented by the Operations Department of hospital based on monthly inspection workload; ③ provide inspectors with the opportunity to receive annual follow-up training related to IPC and participate in emergency drills.

Level I (Internal self-check) consists of 116 self-inspectors. At least one self-inspector was recruited by the HAI management department for each clinical, medical technology or administrative security department. There were a total of 3 supervision forms that need to be completed within one week, covering the development of emergency plans, nucleic acid screening of patients and their escorts at the time of admission, the health Quick Response (QR) code management of escort and visit, the management of infectious disease reports, the management of ward access, the strengthening management of clean areas in wards, HAI prevention and control, the PPE management, training management and logistics support management. The inspection frequency was once a day. The developed mobile phone application (APP) was used to upload the inspection results within one week.

**Fig. 2:**
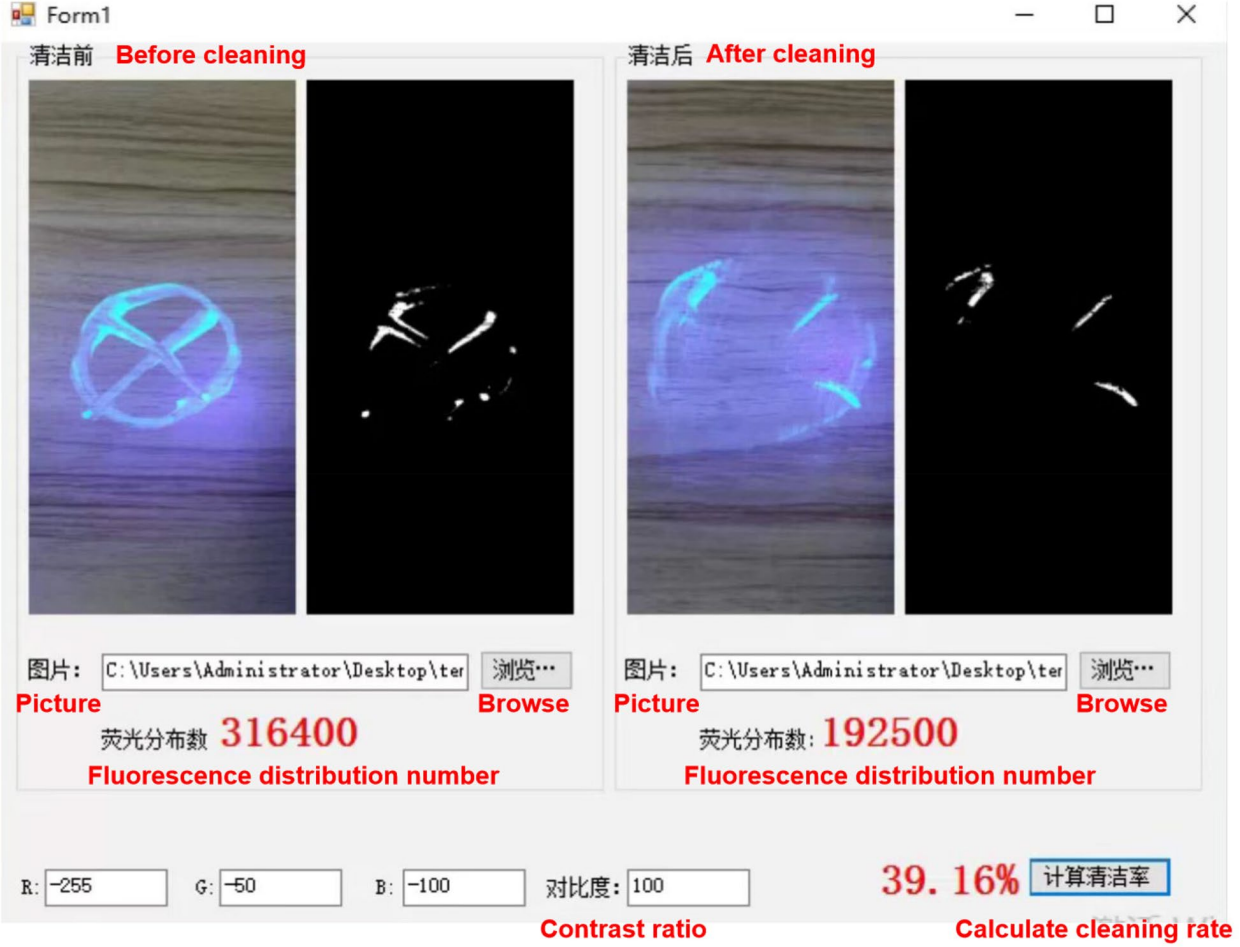
Schematic diagram of artificial intelligence determination of UV/F clearance rate. This artificial intelligence judgment system automatically determine the UV/F clearance rate before and after cleaning

Level II (Departmental cross-check) consists of 63 cross-departmental inspectors. Inspectors at this level were uniformly recruited by the HAI Management Committee and publicized throughout the hospital. The inspection content included the implementation of the emergency measures and the inspection status of the level 1 self-inspector. The inspection objects and items were temporarily released by the HAI management department at the beginning of each month. The inspection frequency was once a week. The cross-departmental inspectors used the developed mobile phone APP to upload the results and immediately feedback to the person in charge of the relevant department the problems found during the cross-inspection process. In addition, ultraviolet light-detectable fluorescent (UV/F) surface marker was included as a mandatory monthly completion project, and artificial intelligence (AI) judgment system based on computer vision was used to calculate the clearance rate. This system operation diagram was shown in Fig. 2, and its intelligent judgment logic was as follows: ① calculate the dot matrix of UV/F brightness pixels through image enhancement, inversion, and binarization processing; ② AI algorithm automatically determine the UV/F clearance rate before and after cleaning, and ultimately generate the rate for each area.

Level III (Verification of outstanding key and difficult issues) consists 12 full-time HAI management staff. The inspection task at this level was to focus on the key and difficult points found in the self- and cross-inspection. The inspectors organized a special team and concentrate resources to solve the problem, and reported the results to the HAI Management Committee.

### Criteria for the selection of inspectors

Both the self- and cross-inspector needed to have some experience in the HAI management to ensure that he/she was able to perform a real supervisory role. In order to enable the inspectors to accurately understand the terms of each inspection, we have provided a corresponding instructional video for each inspection list in the mobile phone APP. Otherwise, given the basic training would be time-consuming [18, 19], the recruited inspectors must meet the following criteria: Internal self-checker must be a member of the daily IPC group, have at least 2 years of clinical experience, and completed the training and assessment for Level I. In addition, an appointed and signed by department leaders for confirmation were wanted. Ultimately 116 self-checkers were included for filing. Departmental cross-checker must be a member of the daily IPC group, have at least 5 years of clinical experience, and completed the training and assessment for Level II. Furthermore, in special departments such as ICU and fever clinics, inspectors must be served by at least one doctor. After screening, 54 cross-checkers were awarded certificates and announced throughout the hospital.

### Definitions and outcomes

Primary outcomes were the execution of the three-level inspector mechanism and compliance with various IPC measures, consisting of PPE compliance of medical personnel, clearance rate of UV/F surface markers and compliance with comprehensive epidemic IPC projects.

According to the Personal Protection Guidelines of Specific Groups during COVID-19 Epidemic, PPE compliance of medical personnel was defined as “PPE usage” and “Wearing and removing process of PPE” [20]. PPE usage was mainly checked for insufficient PPE usage or excessive PPE usage, while wearing and removing process of PPE was to verify whether medical personnel have made the following step errors related to PPE donning/doffing: ① incorrect steps for mask donning; ② incorrect steps for mask doffing; ③ incorrect steps for isolation clothes donning; ④ incorrect steps for isolation clothes doffing; ⑤ incorrect steps for medical protective clothes donning; ⑥ incorrect steps for medical protective clothes doffing; ⑦ incorrect steps for the sequence of PPE donning; ⑧ incorrect steps for the sequence of PPE doffing; ⑨ Hand hygiene was not implemented during the PPE donning/doffing.

Clearance rate of UV/F surface markers involves UV light-visible markers (cl*inell*, GAMMA) on surfaces before terminal cleaning, with assessment post-cleaning of any remaining marks.

According to the technical guidelines for the emergency IPC during COVID-19 epidemic, comprehensive epidemic IPC projects involves department management, cleaning/disinfection, ward management, medical staff protection, medical waste disposal, medical fabric disposal, accompanying and boiling water room management [15–17].

### Data collection

An independent mobile phone APP called “Star of IPC”, which were developed by the healthcare-associated infections quality control center in Sichuan province, were used to realize paperless information transmission and data management. Each inspector in this system was granted an account and privileges that match their inspection level.

### Statistical analysis

Statistical analysis of the data was performed using SPSS 19.0 software. Data were presented as mean [standard deviation (SD)] or frequency [corresponding proportion (%)], for normally continuous and categorical variables respectively. Using one-way ANOVA for continuous variables and the χ^2^ test for categorical variables, data were compared between inspection levels. All tests were 2-sided with an  *α *level of 0.05.

## Results

During the present study period, there were already 2,800,132 supervision records on our dedicated mobile phone APP, including 149,137 comprehensive epidemic IPC projects, 1,410,093 PPE use, 1,223,595 wearing and removing process of PPE and 17,307 UV/F surface markers.

**Fig. 3:**
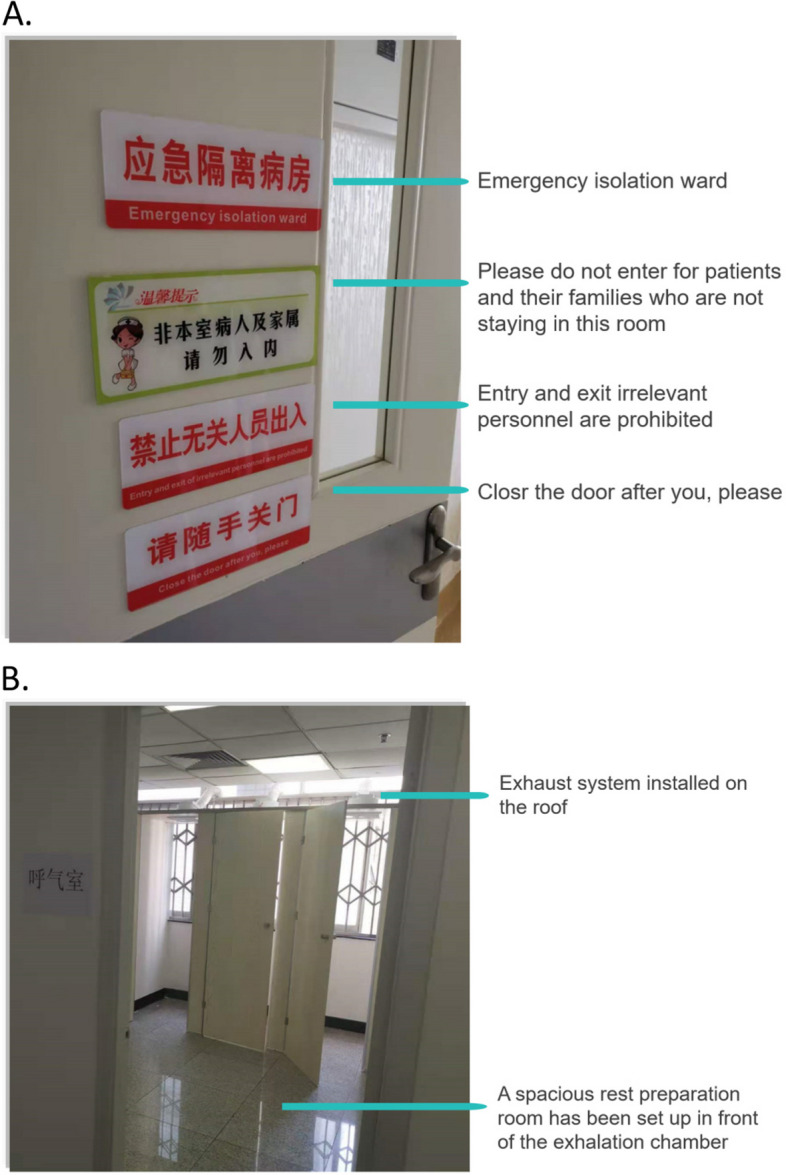
Optimized IPC measures explored by the inspectors and subjects. **A** Warning signs of emergency isolation ward include four contents as follows: ① Emergency isolation ward, ② Please do not enter for patients and their families who are not staying in this room, ③ Entry and exit irrelevant personnel are prohibited, ④ Close the door after you, please; **B** Each independent exhalation chamber can accommodate one person for C-13 urea exhaled gas test. The exhaust system installed on the roof ensures that the exhalation chamber is at a relative negative pressure. These designs have avoided virus transmission due to possible aerosols

The inspection results of comprehensive epidemic IPC projects are shown in Table 1. The compliance rate of check items has exceeded 98%, and internal self-check has a statistically significant higher rate than departmental cross-check (99.95% versus 98.74%, *χ*^2^ = 26111.479, *P* < 0.001). During the study period, the inspectors and subjects explored many optimized IPC measures, such as warning signs of emergency isolation ward (Fig. 3A) and negative pressure chamber prepared for C-13 urea exhaled gas test (Fig. 3B).

**Table 1:** Inspection results of comprehensive epidemic IPC projects

Check items		Internal self-check		Departmental cross-check	
		**Number of valid checks**	**Compliance rates**	**Number of valid checks**	**Compliance rates**
Department management	Formulate the emergency plan and workflow for COVID-19, and timely adjust and improve them according to the prevention and control requirements and actual situation.	136,983	100.00%	11,937	99.70%
	All staff have completed the training of IPC knowledge.	136,937	99.98%	11,986	99.08%
	At least one complete set of PPE shall be provided, including medical surgical masks, particulate respirators, isolation clothing and goggles.	117,404	100.00%	11,956	99.67%
	Define the division of clean area, semi-polluted area and polluted area, establish the concept of sanitation permit for each section (at least hand hygiene), and prohibit wearing any PPE (except clean masks) into the clean area.	136,385	99.89%	11,737	98.99%
Cleaning and disinfection	Check the cleaning and disinfection records of the surface of environmental objects (including the general ward and emergency isolation ward) and the head nurse's inspection records. The above records should be filled in timely and completely.	136,554	100.00%	11,749	98.48%
	The ward environment should be ventilated and air disinfected. Check the air disinfection records.	114,123	100.00%	11,866	99.03%
	Ask the cleaning workers about the disinfectant configuration. Check the configuration of the on-site inspection concentration test paper.	136,575	99.98%	11,430	99.20%
Ward management	The emergency isolation ward shall be set up at the end of the ward to avoid pollution to the surrounding ward.	64,996	99.94%	9,085	98.10%
	Establish relevant work systems and procedures for emergency isolation ward.	68,889	99.99%	9,308	98.69%
	The emergency isolation ward shall be equipped with sufficient disinfection and PPEs for COVID-19.	67,317	99.99%	9,302	98.45%
	The emergency isolation ward should be in the charge of special personnel, and the instruments needed for diagnosis and treatment should be dedicated.	64,722	99.99%	9,148	99.26%
	Implement strict management of accompanying and visiting, so as to minimize the number of accompanying and visiting personnel.	72,784	100.00%	9,551	99.62%
	Strictly manage nucleic acid sampling in the ward, including preparing materials in advance, turning off the air conditioner, closing the door and hanging warning signs, dispersing irrelevant personnel, and using PPEs correctly.	43,693	99.86%	7,780	100.00%
Medical staff protection	On the basis of standard prevention, reasonably use PPEs: ⑴ Work clothes, hats, shoes, surgical masks and quick-drying hand disinfectants should be widely used. ⑵ When collecting respiratory tract samples, endotracheal intubation, tracheotomy, non-invasive ventilation, sputum aspiration and other operations that may produce aerosols, wear work clothes, caps, particulate respirators, protective clothing, latex gloves, and comprehensive respiratory protective devices.	136,413	100.00%	11,621	98.52%
	Properly wear PPEs, especially earloop face mask, which should be tied on the belt to tightly fit the face and avoid falling off during work.	136,592	99.99%	11,695	98.73%
	Supervise and guide medical personnel to wear and take off PPE.	116,628	100.00%	11,206	99.53%
	It is forbidden to leave the contaminated area with PPE.	112,538	99.81%	11,038	99.74%
	Hand hygiene shall be carried out in accordance with the Code of Hand Hygiene for Medical Personnel.	136,769	100.00%	11,788	98.35%
	Strictly implement the preventive measures for sharp injuries.	114,263	99.66%	11,628	99.54%
Medical waste disposal	Wastes (including medical waste and domestic garbage) generated by COVID-19 patients and suspected patients should be collected by category.	24,065	99.78%	5,112	100.00%
	COVID-19 medical waste shall be labeled after separate classification and collection, and the label shall include the medical waste production department, production date, category, weight, and the name of the cleaner. And make account records.	25,365	99.79%	5,119	100.00%
	A special storage area for COVID-19 medical waste should be set up and warning signs should be hung to avoid mixing with conventional medical waste. If the waste is stored separately in the original waste storage room, there should be a clear separation from the conventional medical waste (such as physical partition, aisle.)	36,836	99.88%	5,795	97.38%
	The temporary storage of medical waste should be able to be closed tightly.	59,881	99.81%	6,624	98.61%
	The storage time of COVID-19 medical waste shall not exceed 24 h.	27,108	99.97%	5,022	100.00%
	Assign special personnel to disinfect the temporary storage place of COVID-19 medical waste with 1000 mg/L chlorine containing disinfectant at least twice a day.	33,406	99.97%	5,391	99.02%
	COVID-19 medical waste and conventional medical waste shall fill in the transfer form separately, and register and handover layer by layer.	53,116	99.77%	5,613	99.22%
	PPEs used by medical waste transport personnel shall include medical surgical masks/particulate respirators, disposable hats, latex gloves, isolation clothing or protective clothing, goggles/protective screens, and waterproof boots.	54,259	99.96%	6,111	100.00%
	After delivery, use 1000 mg/L chlorine disinfectant to clean and disinfect the delivery tools.	35,296	99.98%	5,849	99.91%
Medical fabric disposal	Clothes, bedding and other textiles used by COVID-19 patients and suspected patients shall be collected at the bedside in a closed manner to avoid aerosol generation.	22,283	99.84%	4,127	99.98%
	When the clothes, bedding and other textiles used by COVID-19 patients and suspected patients have no obvious blood and body fluid pollution, the double-layer yellow packaging bags shall be used to contain medical fabrics, and then sealed and pasted with labels (marked with "COVID-19 fabric"). After the outer surface of the packaging bag is sprayed with 1000 mg/L chlorine containing disinfectant, it shall be stored in a sealed transfer box (marked with "COVID-19 fabric") in a separate area of the temporary storage room. In case of obvious blood and body fluid pollution, it shall be treated as medical waste.	34,520	99.89%	4,785	99.29%
Accompanying management	Whether it is really implemented to match a patient with a companion.	79,430	99.96%	9,202	97.58%
	Whether the basic information such as the company's identity is complete, including QR code, daily temperature and epidemiological information.	80,096	100.00%	9,240	98.50%
	It is forbidden for accompanying personnel to gather in the ward.	80,955	100.00%	9,259	98.05%
	Urge the company to wear masks.	81,691	99.98%	9,376	96.09%
Boiling water room management	Set warning signs in the boiling water room to avoid crowding.	118,405	99.99%	10,248	95.47%
	Quick-drying hand disinfectants shall be placed in the boiling water room, and signs shall be pasted to prompt the implementation of hand hygiene.	119,357	99.95%	10,338	95.41%
	Urge the cleaning personnel to clean and disinfect the boiling water room every day.	119,476	99.96%	10,360	99.22%
Total		3,136,110	99.95%	333,382	98.74%

**Fig. 4:**
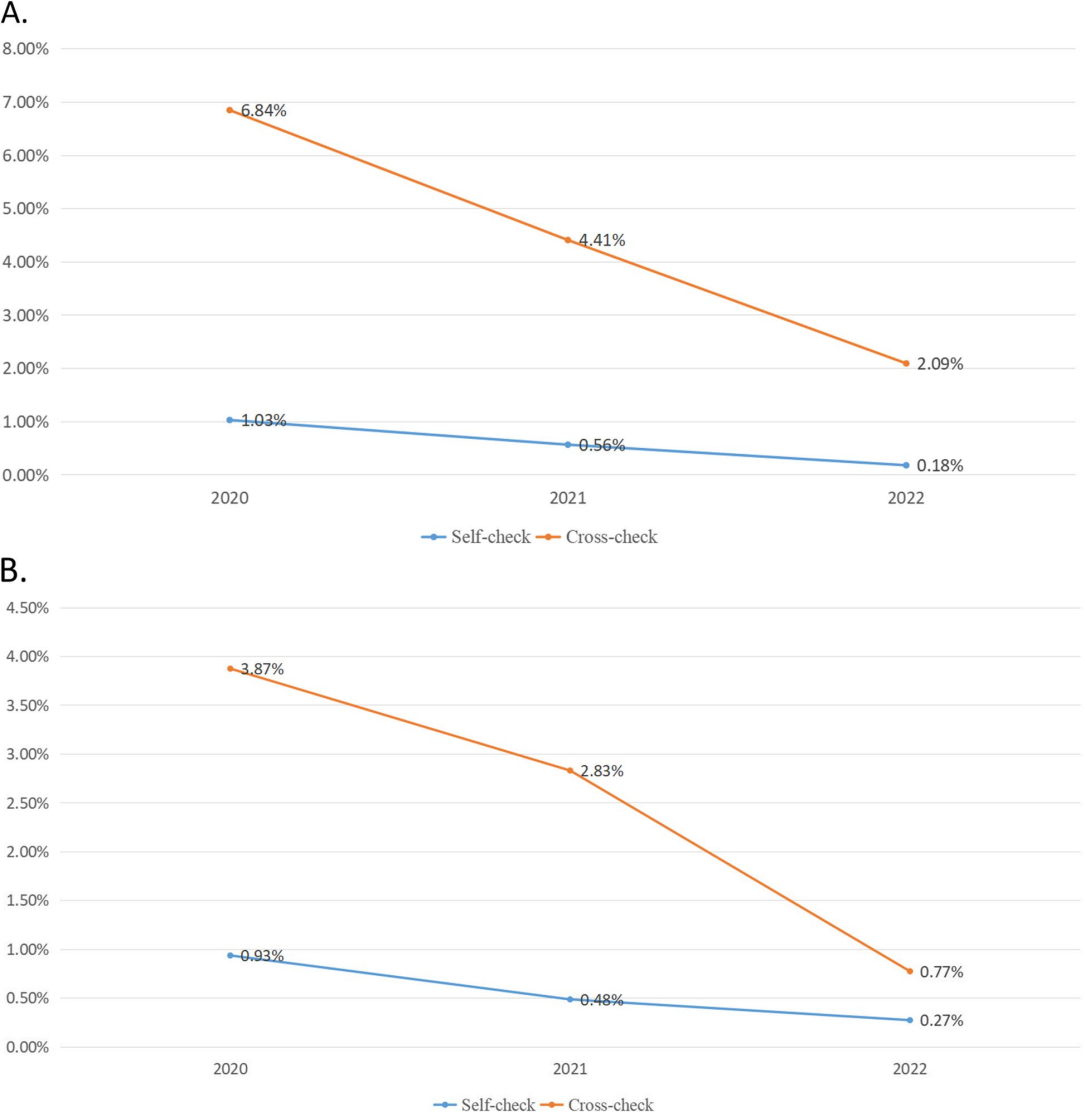
Failure rates show a decreasing trend year by year in both self-check and cross-check. **A** The failure rate of PPE usage was higher in departmental cross-check than in internal self-check; **B** The failure rate of PPE wearing and removing process was higher in departmental cross-check than in internal self-check

94% of all inspection records are contributed by relevant PPE, as show in Table 2. The failure rate of PPE usage was statistically higher in departmental cross-check than in internal self-check (4.17% versus 0.70%, *χ*^2^ = 1957.987, *P* < 0.001). The failure rate of PPE wearing and removing process was statistically higher in departmental cross-check than in internal self-check (2.48% versus 0.64, *χ*^2^ = 465. 610, *P* < 0.001). Of note, these failure rates show a decreasing trend year by year in both self-check and cross-check (*χ*^2^ = 1476.114, *P* < 0.001, *χ*^2^ = 1098.832, *P* < 0.001, *χ*^2^ = 86.565, *P* < 0.001, *χ*^2^ = 50.620, *P* < 0.001, respectively). Figure 4 shows the details.

**Table 2:** Inspection results of PPE related

Year	Levels	PPE usage			Wearing and removing process of PPE		
		**Failure Rates**	**Failures/Total checks**	***P***	**Failure Rates**	**Failures/Total checks**	***P***
2020	Self-check	1.04%	5,374/524,226	< 0.001	0.94%	4,315/461,734	< 0.001
	Cross-check	7.35%	169/2,469		4.03%	84/2,169	
2021	Self-check	0.57%	4,234/752,688	< 0.001	0.49%	3,155/651,262	< 0.001
	Cross-check	4.61%	249/5,652		2.91%	117/4,136	
2022	Self-check	0.18%	213/121,228	< 0.001	0.27%	276/101,704	< 0.001
	Cross-check	2.13%	80/3,830		0.78%	20/2,590	
Total	Self-check	0.70%	9821/1398142	< 0.001	0.64%	7746/1214700	< 0.001
	Cross-check	4.17%	498/11951		2.48%	221/8895

The overall clearance rate of UV/F surface markers is 87.88%. As shown in Table 3, there was no statistically significant difference in clearance rate of UV/F surface markers over the three years of the present study (*F* = 2.902, *P* = 0.071).

**Table 3:** Clearance rate of UV/F surface markers

Month	2020	2021	2022
January	/	82.83%	85.59%
February	/	84.36%	89.84%
March	/	82.88%	83.90%
April	/	80.13%	87.60%
May	99.03%	82.78%	97.57%
June	97.05%	86.06%	100.00%
July	97.62%	88.11%	100.00%
August	88.29%	83.50%	90.71%
September	85.43%	85.01%	80.27%
October	83.17%	90.64%	90.10%
November	84.73%	89.38%	91.20%
December	80.09%	88.59%	92.40%
*F*	2.902		
*P*	0.071		

## Discussion

This study found that three irreplaceable functions reflect the effectiveness of the inspector mechanism. On the one hand, the implementation of daily inspections made it possible for maintaining the best IPC status at any time. On the other hand, the awareness and enthusiasm of IPC has been maximized because the common threat of COVID-19 promoted focus and unity [21]. Many optimized IPC measures innovated by the inspectors and subjects demonstrate that the entire hospital IPC culture was sublimated. In addition, in response to the COVID-19 challenge, IPC inspections, which were previously considered a burden, have now become a means of protection for medical staff [22]. Like the observing system of Guangdong Second Provincial General Hospital, our inspector mechanism also is an infection control system to provide real-time monitoring and aid for instant correction so as to minimize the HAI risk [11]. Studies indicated that inspectors mechanism in increasing compliance with PPE use and reducing COVID-19 infection spread was widely considered to be effective [10, 23]. Of note, our part-time inspector system expanded the content and scope of supervision on the basis of previous studied.

In the present study, the appropriate usage of PPE which has been improved year by year suggested that enforcement strategy through the three-level inspector mechanism have positive attributes. With the continuous change of knowledge about COVID-19 during the crisis, the PPE usage and the proper doffing/donning technology were widely considered to be crucial, but also vague and inconsistent [24]. Even though the available guidelines for reference were updated quickly in intensity with the ongoing crisis, medical staff still struggled to choose PPE reasonably according to specific job positions [25]. The role of the inspection mechanism was not only reflected in increased PPE compliance, but more importantly, it unified proper awareness amongst staff.

Based on the clear UV/F utility of previous studies [26], we attempted to directly improve cleaning compliance using UV/F inspection method. Referring to Matthew's 20% failure rate, we believed that a clearance rate of over 80% was acceptable [27]. Notably, an AI tool was used to avoid observer bias caused by variant subjective perceptions. However, the validity in application of UV/F markers has not been defined in this study due to the lack of parallel control. Consistent with previous studies [26, 27], even with well-equipped quality control tools for the removal of the UV/F marker, the clearance rate of UV/F markers over the study period was not statistically significant. The variability in application of UV/F markers seems to make it difficult to translate into effective cleaning overall, which needs to be continuously explored in the future.

To stimulate stronger initiative, it was very necessary to configure some basic safeguards, such as sufficient supervision time and remuneration [28]. We required every department leader to sign a letter of commitment, which mainly included serving as the first person responsible for the supervision of their own department, promising to give the supervisor half a day a week to complete the task and providing supervisors with an opportunity for education or training related to HAI management once a year [29]. In terms of remuneration, our hospital had allocated an annual budget of US$30,000 for part-time HAI management. Most notably, considering it was easy to get tired of complicated and boring supervision work, quality control for supervision was an essential part [30]. A feasible method was recommended to link the supervision quantity and quality with monthly remuneration, so as to better performance of their duties could be guaranteed through incentive mechanism [31].

Judging from the records number of supervision results, the three-level inspector mechanism had well alleviated the complex basic supervision work of full-time staff, who could screen out key problems and links from these results, rather than went to the ward to slowly search for risk clues. Given that the professional quality of part-time inspectors could not reach the level of full-time staff, quantitative terms were recommended for widely use in supervision items [32]. Compared with quantitative terms, subjective judgment terms had higher requirements for inspectors' knowledge reserve. Simple and easy to grasp supervision methods could significantly improve efficiency and achieve remarkable results.

The mobile phone APP called "Star of IPC", which had been already certified by the National Copyright Administration of the People's Republic of China, had realized a paperless network platform so as to avoid cross-contamination through paper [33]. In Sichuan Province, China, 629 medical institutions have used this system for COVID-19 IPC. However, the prominent difficulty we encountered was that the APP review process for the public release was too long. Shorter frequency of APP version updates were recommend as much as possible, and it was best to design the APP function and content comprehensively in advance.

## Strengths and limitations

To our knowledge, this is the largest study to investigate the effectiveness of inspector mechanism for the emergency IPC in COVID-19 epidemic period. The main strengths of this paper include continuous intervention lasting for 3 years, extensive supervisory records, paperless office inspection and AI tools.

A number of limitations of this study should be considered. First, the self-control study design have not accounted for other confounding factors that may have led to imbalance. Second, due to sporadic COVID-19 epidemic under extreme IPC, the evaluation of systemic effects was limited in the cluster levels rather than individual disease levels, which needs to be further explored.

## Conclusion

Inspector mechanism for the emergency IPC in COVID-19 epidemic period from 2020 to 2022 completed an incredible inspection workload and offered creative assistance to combat the COVID-19 outbreak. Although there were some difficulties in the implementation process, optimization methods could gradually be explored to make the system run effectively. These methods and accumulated experiences should be helpful for us to strengthen IPC for future epidemic.

### Supplementary Information

**Additional file 1: SM Table S1. **Details of intervention units.

## Data Availability

The datasets used during the present study are available from the corresponding author on reasonable request.
