# Supplementary material for: Effectiveness of inspector mechanism for the emergency infection prevention and control in the SARS-CoV-2 epidemic period: a self-control real-word study
Source: BMC Infect Dis. 2023 Dec 6;23:858. doi: 10.1186/s12879-023-08682-2 (PMC10702026; doi:10.1186/s12879-023-08682-2)
Supplement: Supplementary file 1 — Additional file 1: SM Table S1. Details of intervention units. [file 12879_2023_8682_MOESM1_ESM.docx]

**SUPPLEMENTAL MATERIAL**

***Effectiveness of inspector mechanism for the emergency infection prevention and control in the SARS-CoV-2 epidemic period: a self-control real-word study***

*Yu Lv^(a1)^, Qian Xiang^(a1)^, Xiaoyan Jiang^(a1)^, Bo Zhang^(a3)^, Jiayu Wu^(a1)^, Hongrong Cao^(a1)^.*

*a1: Healthcare-associated Infection Control Center, Sichuan Academy of Medical Sciences, Sichuan People's Hospital, School of Medicine, University of Electronic Science and Technology of China, Chengdu, 610072, Sichuan, P. R. China.*

*a2: Department of Nursing, Sichuan Academy of Medical Sciences, Sichuan People's Hospital, School of Medicine, University of Electronic Science and Technology of China, Chengdu, 610072, Sichuan, P. R. China.*

*a3: Development Department, Chengdu Yiou Technology co. LTD, Chengdu, 610000, Sichuan, P. R. China.*

**TABLES**

**SM Table S1. Details of intervention units**

| **Department** | **Type** | **Locale** |
| --- | --- | --- |
| Hematology,respiratory department in Caotang Hospital Area | Clinical | Caotang Hospital Area |
| Geriatric Internal Medicine Department in Caotang Hospital Area | Clinical | Caotang Hospital Area |
| Gastroenterology Department in Caotang Hospital Area | Clinical | Caotang Hospital Area |
| Emergency Internal Medicine Department | Clinical | Emergency treatment 3F |
| Emergency surgery | Clinical | Emergency treatment 5F |
| Burn wound repair department | Clinical | Emergency treatment 6F |
| Elderly Digestive Department | Clinical | Hospital building number five 1F |
| Elderly Infection Department | Clinical | Hospital building number five 1F |
| Geriatric Endocrinology Department | Clinical | Hospital building number five 2F |
| Neurology for the Elderly | Clinical | Hospital building number five 2F |
| Geriatric Respiratory Department | Clinical | Hospital building number five 3F |
| Elderly vascular department 2 | Clinical | Hospital building number five 4F |
| Geriatric Internal Medicine Department 2 | Clinical | Hospital building number five 5F |
| Urology Surgery 1 | Clinical | Hospital building number one 10F |
| Urology Surgery 2 | Clinical | Hospital building number one 10F |
| Robot center | Clinical | Hospital building number one 11F |
| Plastic surgery | Clinical | Hospital building number one 11F |
| Ophthalmology 1 | Clinical | Hospital building number one 12F |
| Ophthalmology 2 | Clinical | Hospital building number one 12F |
| Organ Transplantation Center 1 | Clinical | Hospital building number one 13F |
| Organ Transplantation Center 2 | Clinical | Hospital building number one 13F |
| Gynecology 1 | Clinical | Hospital building number one 14F |
| Cell Transplantation Center | Clinical | Hospital building number one 14F |
| Gastrointestinal surgery 1 | Clinical | Hospital building number one 15F |
| Gastrointestinal surgery 2 | Clinical | Hospital building number one 15F |
| Gynecology 2 | Clinical | Hospital building number one 16F |
| Gynecology 3 | Clinical | Hospital building number one 16F |
| Cardiac surgery 1 + Heart Failure Center | Clinical | Hospital building number one 17F |
| Cardiac surgery 2 | Clinical | Hospital building number one 17F |
| Obstetrical department 2 | Clinical | Hospital building number one 5F |
| Obstetrical department 3 | Clinical | Hospital building number one 5F |
| Delivery Room | Clinical | Hospital building number one 6F |
| Maxillofacial | Clinical | Hospital building number one 6F |
| Thoracic surgery 2 | Clinical | Hospital building number one 8F |
| Thoracic surgery 1 | Clinical | Hospital building number one 8F |
| Hepatology 1 | Clinical | Hospital building number one 9F |
| Hepatology 2 | Clinical | Hospital building number one 9F |
| Otolaryngology Head and Neck Surgery | Clinical | Hospital building number three 10F |
| Pediatric Surgery | Clinical | Hospital building number three 11F |
| Department of nephrology 1 | Clinical | Hospital building number three 12F |
| Department of nephrology 2 | Clinical | Hospital building number three 13F |
| Neurosurgery 1 | Clinical | Hospital building number three 14F |
| Neurosurgery 2 | Clinical | Hospital building number three 15F |
| Orthopedics 1 | Clinical | Hospital building number three 16F |
| Orthopedics 2 | Clinical | Hospital building number three 17F |
| Breast Surgery | Clinical | Hospital building number three 19F |
| Vascular Surgery | Clinical | Hospital building number three 20F |
| Geriatric cardiovascular 3 | Clinical | Hospital building number three 21F |
| Geriatric cardiovascular 1 | Clinical | Hospital building number three 21F |
| Geriatric Internal Medicine Department 3 | Clinical | Hospital building number three 22F |
| Geriatric Internal Medicine Department 1 | Clinical | Hospital building number three 23F |
| Dermatology | Clinical | Hospital building number three 4F |
| Comprehensive Rehabilitation Department 1 | Clinical | Hospital building number three 6F |
| Hyperbaric Oxygen Department | Clinical | Hospital building number three 8F |
| Pain department | Clinical | Hospital building number three 8F |
| Orthopedic rehabilitation | Clinical | Hospital building number three 9F |
| Neurology 1 | Clinical | Hospital building number two 10F |
| Neurology 2 | Clinical | Hospital building number two 11F |
| Internal Medicine-Neurology 3 | Clinical | Hospital building number two 12F |
| Oncology department 1 | Clinical | Hospital building number two 13F |
| Oncology department 2 | Clinical | Hospital building number two 14F |
| Rheumatology and Immunology Department | Clinical | Hospital building number two 15F |
| Cardiology Department 1 + CCU | Clinical | Hospital building number two 16F |
| Cardiology Department 2 | Clinical | Hospital building number two 17F |
| Respiratory medicine 1 | Clinical | Hospital building number two 18F |
| Respiratory failure center and Lung transplantation ward | Clinical | Hospital building number two 19F |
| Gastroenterology | Clinical | Hospital building number two 20F |
| Endocrinology department | Clinical | Hospital building number two 21F |
| Blood specialty | Clinical | Hospital building number two 22F |
| Department of Infectious Diseases | Clinical | Hospital building number two 2F |
| General ward | Clinical | Hospital building number two 5F |
| Pediatrics 1 | Clinical | Hospital building number two 6F |
| TCM department | Clinical | Hospital building number two 7F |
| Department of pediatrics 2 | Clinical | Hospital building number two 9F |
| Emergency ICU | ICU | Emergency treatment 2F |
| Extracardiac monitoring room | ICU | Hospital building number one 17F |
| SICU | ICU | Hospital building number one 7F |
| Elderly intensive care unit | ICU | Hospital building number three 18F |
| Neurosurgery ICU | ICU | Hospital building number three 5F |
| Internal Medicine-Neurology ICU | ICU | Hospital building number two 12F |
| Medical ICU | ICU | Hospital building number two 19F |
| Neonatology | ICU | Hospital building number two 8F |
| Pediatric Care Unit | ICU | Hospital building number two 9F |
| Blood Transfusion Department | Medical skill | Hospital building number one 1F |
| Health Management Center | Medical skill | Hospital building number three |
| Dermatology laser room | Medical skill | Hospital building number three 4F |
| Medical record department | Medical skill | Hospital building number two 23F |
| Radiology department | Medical skill | Outpatient and emergency |
| Department of Ultrasound | Medical skill | Outpatient and emergency |
| Cardiovascular ultrasound department and cardiac function room | Medical skill | Outpatient service 4F |
| Stomatology Clinic | Medical skill | Outpatient service 4F |
| Department of Pathology | Medical skill | Outpatient service 5F |
| Endoscopy Center in Caotang Hospital Area | Outpatient service | Caotang Hospital Area |
| Emergency examination triage + emergency treatment | Outpatient service | Emergency treatment 1F |
| Hospital building number three | Outpatient service | Hospital building number three |
| TCM Clinic | Outpatient service | Hospital building number three |
| Prevention & Healthcare Center | Outpatient service | Hospital building number three outpatient service |
| PICC Management and Maintenance Center | Outpatient service | Hospital building number three outpatient service |
| Dermatology Department Outpatient Department | Outpatient service | Hospital building number three outpatient service |
| Obstetrics and Gynecology Clinic + Family Planning Operating Room | Outpatient service | Hospital building number two outpatient service |
| Outpatient Ophthalmology (Myopia center) | Outpatient service | Hospital building number two outpatient service |
| Outpatient Injection,Emergency injection room | Outpatient service | Outpatient and emergency |
| Outpatient Wound Care Center (Dressing change area) | Outpatient service | Outpatient and emergency |
| Outpatient department 3F,4F | Outpatient service | Outpatient service |
| Outpatient child care 2F | Outpatient service | Outpatient service |
| Pediatric Observation Room + pediatric clinic | Outpatient service | Outpatient service and pediatrics |
| Outpatient oncology | Outpatient service | Outpatient service1F |
| Otolaryngology Clinic and decontamination chamber | Outpatient service | outpatient service3F |
| Interventional Center | Special department | Hospital building number three 4F |
| Hemodialysis Center | Special department | Hospital building number three 5F |
| Three bedroom operating room | Special department | Hospital building number three 6F |
| Disinfection Supply Center | Special department | Hospital building number two -1F |
| Operating room | Special department | Operating room |
| Clinical Laboratory + Nuclear Medicine Department | Special department | Outpatient service 3F |
| Endoscopy room | Special department | Outpatient service 7F (old library) |
